# Temporal patterns in *Ixodes ricinus* microbial communities: an insight into tick-borne microbe interactions

**DOI:** 10.1186/s40168-021-01051-8

**Published:** 2021-07-03

**Authors:** E. Lejal, J. Chiquet, J. Aubert, S. Robin, A. Estrada-Peña, O. Rue, C. Midoux, M. Mariadassou, X. Bailly, A. Cougoul, P. Gasqui, J. F. Cosson, K. Chalvet-Monfray, M. Vayssier-Taussat, T. Pollet

**Affiliations:** 1 UMR BIPAR, Animal Health Laboratory, INRAE, ANSES, Ecole Nationale Vétérinaire d’Alfort, Université Paris-Est, Maisons-Alfort, France; 2 Université Paris-Saclay, AgroParisTech, INRAE, UMR MIA-Paris, 75005 Paris, France; 3 Faculty of Veterinary Medicine, University of Zaragoza, Zaragoza, Spain; 4 INRAE, MaIAGE, Université Paris-Saclay, Jouy-en-Josas, France; 5 INRAE, BioinfOmics, MIGALE Bioinformatics Facility, Université Paris-Saclay, Jouy-en-Josas, France; 6 INRAE, PROSE, Université Paris-Saclay, Antony, France; 7 Université Clermont Auvergne, INRAE, VetAgro Sup, UMR EPIA, 63122 Saint Genes Champanelle, France; 8 Université de Lyon, INRAE, VetAgro Sup, UMR EPIA, 69280 Marcy l’Etoile, France; 9 INRAE, Animal Health Department, Nouzilly, France; 10 UMR ASTRE, CIRAD, INRAE, Campus de Baillarguet, Montpellier, France

## Abstract

**Background:**

Ticks transmit pathogens of medical and veterinary importance and are an increasing threat to human and animal health. Assessing disease risk and developing new control strategies requires identifying members of the tick-borne microbiota as well as their temporal dynamics and interactions.

**Methods:**

Using high-throughput sequencing, we studied the *Ixodes ricinus *microbiota and its temporal dynamics. 371 nymphs were monthly collected during three consecutive years in a peri-urban forest. After a Poisson lognormal model was adjusted to our data set, a principal component analysis, sparse network reconstruction, and differential analysis allowed us to assess seasonal and monthly variability of *I. ricinus* microbiota and interactions within this community.

**Results:**

Around 75% of the detected sequences belonged to five genera known to be maternally inherited bacteria in arthropods and to potentially circulate in ticks: *Candidatus* Midichloria, *Rickettsia*, *Spiroplasma*, *Arsenophonus* and *Wolbachia*. The structure of the *I. ricinus* microbiota varied over time with interannual recurrence and seemed to be mainly driven by OTUs commonly found in the environment. Total network analysis revealed a majority of positive partial correlations. We identified strong relationships between OTUs belonging to *Wolbachia* and *Arsenophonus*, evidence for the presence of the parasitoid wasp *Ixodiphagus hookeri* in ticks. Other associations were observed between the tick symbiont *Candidatus* Midichloria and pathogens belonging to *Rickettsia*. Finally, more specific network analyses were performed on TBP-infected samples and suggested that the presence of pathogens belonging to the genera *Borrelia*, *Anaplasma* and *Rickettsia* may disrupt microbial interactions in *I. ricinus*.

**Conclusions:**

We identified the *I. ricinus* microbiota and documented marked shifts in tick microbiota dynamics over time. Statistically, we showed strong relationships between the presence of specific pathogens and the structure of the *I. ricinus* microbiota. We detected close links between some tick symbionts and the potential presence of either pathogenic *Rickettsia* or a parasitoid in ticks. These new findings pave the way for the development of new strategies for the control of ticks and tick-borne diseases.

Video abstract.

**Supplementary Information:**

The online version contains supplementary material available at 10.1186/s40168-021-01051-8.

## Introduction

Ticks are vectors of many zoonotic pathogens and are an important and increasing threat to human and animal health. While these arthropods are among the main vectors of pathogens that affect humans and animals worldwide, it is now well established that tick-borne pathogens (TBPs) coexist with many other microorganisms in ticks. These other members of the microbiota, commensal and/or symbionts, are likely to confer multiple detrimental, neutral, or beneficial effects to their tick hosts and can play various roles in nutritional adaptation, development, reproduction, defense against environmental stress and immunity [1, 2]. They may also interact with tick-borne pathogens thereby influencing the competence of the tick vector [3, 4]. Identifying and characterising the tick microbiota is thus crucial to better understand tick-microbe interactions. With the development of high-throughput sequencing technologies, the number of studies dealing with tick microbiota has considerably increased in the past decade and revealed unexpected microbial diversity in ticks [5–14]. The microbiota of several tick species belonging to the genera *Ixodes*, *Dermacentor*, *Haemaphysalis*, *Rhipicephalus* and *Amblyomma* has been studied [15], revealing key details on microbial communities in ticks and improving our knowledge of tick microbiota ecology in general. However, while *Ixodes ricinus* is the main tick species present in western Europe, able to transmit the widest range of pathogens (including the agent of Lyme disease, *Borrelia burgdorferi* s.l.), the ecological factors driving variations in its microbiota have been the subject of few studies [16]. Moreover, several of the previous studies of the *Ixodes ricinus* microbiota [6, 12, 16–22], may have overestimated microbiota diversity due to contamination during the extraction step and the absence of negative controls in their analysis [22]. Studies of *I. ricinus* microbiota pointed to complex microbial assemblages inhabiting ticks including pathogens, specific endosymbionts, commensal and environmental microorganisms. As already reported for pathogens [23–26], the other members of these biological assemblages are probably dynamic and likely to vary over time. While the observation scale affects our view of the dynamics of tick microbiota [27], little information is currently available on the temporal patterns of the *I. ricinus* microbiota. Does the *I. ricinus* microbiota vary with the season? Are these potential temporal patterns repeated annually? Answering these questions is a crucial first step to better understand both the tick and tick microbiota ecology. While it is now accepted that tick microbiota may also play a role in driving transmission or multiplication of tick-borne pathogens [28], little information is currently available on the interactions between *I. ricinus*-borne microbiota members and on the potential co-occurrence of pathogens and the *I. ricinus* microbiota [16]. This information is needed to identify potential strategies for the control of ticks and tick-borne diseases in the future. *I. ricinus* nymphs were collected monthly in three consecutive years in a peri-urban forest. High-throughput sequencing was used to identify the *I. ricinus* microbiota and its temporal dynamics. Using multivariate and partial correlation network analyses, the aim of this study was to identify direct statistical associations between members of the microbiota, including pathogenic genera, and to assess the influence of the presence of TBPs on tick microbiota structure and interactions.

## Material and methods

### Tick collection

Questing *Ixodes ricinus* nymphs were collected for 3 years by dragging (from April 2014 to May 2017) in the Sénart forest in the south of Paris. More details on the sampling location and design, and tick collection, are available in Lejal et al. [26].

### Tick homogenisation and DNA extraction

In total, 998 nymphs have been collected over the 3 years [26]. As detailed in our previous studies [26, 29], ticks were first washed once in ethanol 70% for 5 min and rinsed twice in sterile MilliQ water. They were then individually homogenised in 375 μL of Dulbecco’s modified Eagle’s medium with decomplemented foetal calf serum (10%) and six steel beads using the homogeniser Precellys®24 Dual (Bertin, France) at 5500 rpm for 20 s. DNA extraction was performed on 100 μL of tick homogenate, using the NucleoSpin® Tissue DNA extraction kit (Macherey-Nagel, Germany).

### DNA amplification and multiplexing

Among the 998 nymphs, 557 have been chosen to characterise the temporal dynamics of *I. ricinus* microbiota. Thanks to our previous study using microfluidic PCR [26], we knew the infection rate of all collected ticks. For each collecting month, while all infected nymphs have been compulsory integrated in the analysis, non-infected nymphs (at least 15 per month) were added until reaching a minimum of 30 ticks (infected and non-infected) per month, when it was possible. When less than 30 ticks were collected in a month, all ticks were added in the analysis. In addition to tick samples, 45 negative controls have been performed to distinguish tick microbial OTUs from contaminants [22]. As detailed in Lejal et al. [22], DNA amplifications were performed on the V4 region of the 16S rRNA gene using the primer pair used by Galan et al. [30] (16S-V4F: 5′-GTGCCAGCMGCCGCGGTAA-3′ and 16S-V4R: 5′-GGACTACHVGGGTWTCTAATCC-3′), producing a 251-bp amplicon. Different 8 bp indexes were added to primers allowing *in fine* the amplification and multiplexing of all samples. All the PCR amplifications were carried out using the Phusion® High-Fidelity DNA Polymerase amplification kit (Thermo Scientific, Lithuania). For each sample, 5 μL of DNA extract were amplified in a 50 μL final reaction volume, containing 1X Phusion HF buffer, 0.2 μM of dNTPs, 0.2 U/mL of Phusion DNA polymerase and 0.35 μM of forward and reverse primer. The following thermal cycling procedure was used: initial denaturation at 98 °C for 30 s, 35 cycles of denaturation at 98 °C for 10 s, annealing at 55 °C for 30 s, followed by extension at 72 °C for 30 s. The final extension was carried out at 72 °C for 10 min. PCR products were checked on 1.5% agarose gels, cleaned and quantified and finally pooled at equimolar concentrations and sent to the sequencing platform (GenoScreen, France) [22].

### Sequencing and data processing

The equimolar mix was concentrated and sequenced by GenoScreen (Lille, France) using MiSeq Illumina 2 × 250 bp chemistry. All the quality controls and different steps of sequence analyses have been performed (more details are available in [22]). Based on results obtained in the 45 negative controls, sequences considered as contaminants were removed from the dataset [22] (Additional file 1). Due to this large OTU filtration, we identified 186 samples with less than 500 sequences. We considered this number of sequences too low to be analysed and we thus removed these samples. Finally, the microbiota of 371 *I. ricinus* nymphs was analysed from a final dataset composed of 907,941 sequences.

### Data preprocessing

Three distinct datasets have been analysed during this study. First, the whole one, including all the nymphs analysed in 16S rRNA gene sequencing, except the only two nymphs collected in November (369 analysed samples). Second, the No *Wolbachia*/*Arsenophonus* dataset, corresponding to a reduced dataset, where samples harbouring OTUs belonging to *Arsenophonus* and/or *Wolbachia* genera (presenting a number of sequences significantly higher than the highest number of sequences detected for the same OTUs in negative controls, in a 95% confidence interval) were removed (295 samples analysed in this data set). Third, the TBP dataset, composed of samples undoubtedly identified as TBPs positive for only one genus of TBPs (co-infected samples were not included due to the too low number of samples and the difficulty to interpret the results) according to 16S rRNA gene sequencing results as well as microfluidic PCR detection [26]. We considered a sample as positive if a TBP species was previously detected in microfluidic PCR and if at least one OTU identified in 16S rRNA gene sequencing, corresponding to the same pathogenic genera, presented a number of sequences significantly higher than the highest number of sequences detected for this OTU in negative controls (using a 95% confidence interval). Before analysing the data, we applied standard filtering to all the three data sets to remove OTUs associated with weak counts that could hamper the statistical analysis. First, OTUs whose total number of sequences was lower than the total number of samples have been removed. Second, a filter related to the yearly prevalence of each OTU: those consistently detected in less than 10% of the samples of each year were removed. The application of these filters on the three datasets, the whole, the No *Wolbachia*/*Arsenophonus* dataset and the TBPs dataset, led to the selection of 89, 82 and 74 OTUs to be included in the subsequent statistical analyses, respectively.

### Statistical analysis

The statistical framework used to describe our data sets is the multivariate Poisson lognormal (PLN) distribution [31]. This statistical distribution is adapted to multivariate count data and shows an expected over-dispersion property compared to the standard Poisson distribution. The main idea behind the PLN model is to represent all the dependency structure between the OTUs in a latent (hidden) multivariate Gaussian layer, while a Poisson distribution in the observation space of the data is used to model counts and noise. Moreover, the PLN framework can be easily interpreted as a Generalised Multivariate Linear Model (GLM); thus it naturally allows one to include structuring covariates or offsets like in a standard GLM. All the statistical analyses performed in our paper rely on the PLN model and its variants implemented in the R package PLNmodels (version 0.11.0-9005) [32]. In particular, PLNmodels includes variants to perform standard multivariate analyses for count tables, such as PCA (principal component analysis), LDA (linear discriminant analysis) or sparse network reconstruction (aka sparse inference of (inverse) covariance matrix). Additional methodological details can be found in Chiquet et al. [33, 34].

In all the models fitted in this paper, we accounted for the sampling effort (that is, the sequencing depth of each sample) by adding an offset term corresponding to the (log) total sum of counts per sample obtained prior to any filtering. Moreover, in order to correct for any spurious effect induced by a given year of sampling that may hide other effects such as seasonality, we included a covariate to account for the year of sampling in the two first models fitted on both the whole and the No *Wolbachia*/*Arsenophonus* dataset. While investigating the effect of TBPs on tick microbiota, we also systematically included covariates to account for the year and the season of sampling while dealing with the TBP dataset. A covariate accounting for the presence of TBPs was also considered in the establishment of TBP network in order to evaluate the importance of this variable on tick microbiota network establishment.

Principal component analysis was performed with the PCA variant of PLN and the PLNPCA R function [34], which performs probabilistic Poisson PCA. Network analysis was performed with the PLNnetwork function, which adds a sparsity constraint on the inverse covariance matrix in the latent Gaussian layer [33] to reconstruct direct associations. Working from the binary adjacency matrix generated from PLNnetwork, we performed a Bernoulli stochastic block model using the BM Bernoulli function of Stochastic Block Model package [35]. This model is a mixture model for binary graph which assumes that nodes belong to different groups according to their connectivity pattern. The number of groups is selected according an integrated completed likelihood criterion. Thanks to this approach, OTUs presenting similar connection profiles were identified and assigned to different clusters.

A differential analysis was also performed using the edgeR package (version 3.30.0) [36] on the TBPs dataset to compare OTU abundances between positive and negative samples. *edgeR* uses the negative binomial (NB) distribution to model the read counts for each OTU in each sample and computes an empirical Bayes estimate of the NB dispersion parameter for each OTU, with abundance levels specified by a log-linear model. For the same reasons as previously described, we introduced two covariates to control for the year and the season when testing the group main effect (TBP positive or negative). Data were normalised for differences between library sizes using the Trimmed Mean of M value (TMM) method [37]. Models are fitted with the glmFit function, which implements generalised linear methods developed by McCarthy et al*.* [38]. For each OTU, we tested the group effect using likelihood ratio statistics (glmLRT function). Differentially abundant OTUs were defined as those with p values < 0.05 after adjustment for multiple testing using the Bonferroni procedure.

Networks generated from the TBPs dataset were also compared to each other. For this purpose, a weighted version of Kendall’s τ, which integrates the edge appearance rank within families of networks (negative, *Rickettsia*, *Borrelia*, *Anaplasma* and total corrected for TBP effects), was calculated and used to compare them with each other.

All the statistical analyses were performed on R 4.0.2 [39].

## Results

### *Ixodes ricinus* microbiota diversity and composition

**Fig. 1:**
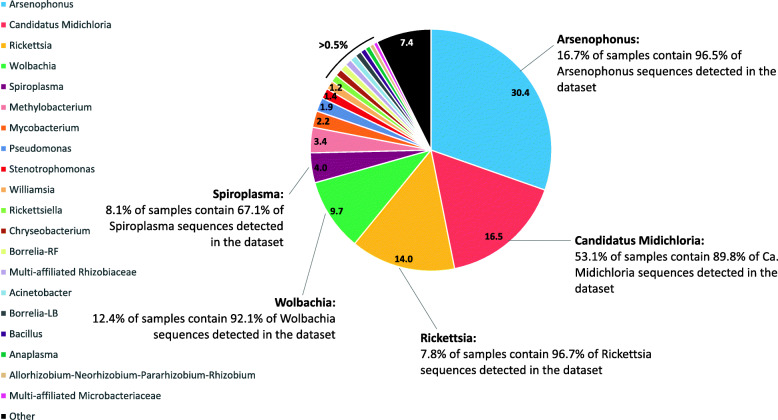
Most dominant genera in the *Ixodes ricinus* microbiota. Selected genera and multi-affiliated OTUs are those representing more than 0.5% of the total number of sequences detected in the whole dataset. Numbers given in the pie chart correspond to this percentage

Considering the microbiota of all the 371 nymphs, we detected 353 OTUs. Among them, 307 belonged to 109 identified genera, and 46 OTUs belonged to multi-affiliated or unknown genera spread over 15 families. The mean Shannon diversity index is estimated to 2.1 (SD = ± 0.8) and varies between 0.3 (nymph collected in April 2016) and 3.8 (nymph collected in October 2014). Bacterial genera with proportions higher than 0.5% of all sequences in the dataset belonged to *Arsenophonus*, *Candidatus* Midichloria, *Rickettsia*, *Wolbachia*, *Spiroplasma*, *Methylobacterium*, *Mycobacterium*, *Pseudomonas*, *Stenotrophomonas*, *Williamsia*, *Rickettsiella*, *Chryseobacterium*, *Borrelia*, *Bacillus*, *Anaplasma*, *Allorhizobium-Neorhizobium-Pararhizobium-Rhizobium* and two multi-affiliated OTUs belonging to Rhizobiaceae and Microbacteriaceae families. In total, these sequences represented 93% of all sequences in the dataset (Fig. 1).

Although these genera represent a large number of sequences identified in the dataset, it is important to note that their presence is not equally distributed in all the samples. To investigate this point, we determined, for the five first represented genera in terms of total number of sequences (*Arsenophonus*, *Ca*. Midichloria, *Rickettsia*, *Wolbachia* and *Spiroplasma*), the number of samples where they had a relative abundance of at least 10%. For *Arsenophonus*, while this genus corresponded to 30% of the total number of sequences in the dataset, we detected sequences of this genus in only 16.7% of samples. These 16.7% of samples contained 96.5% of the total number of sequences in the dataset for this genus. We found the endosymbiont *Ca*. Midichloria, in 53% of nymphs which contained 90% of the total number of *Ca*. Midichloria sequences. Concerning *Rickettsia*, only 7.8% of samples concentrated 97% of the total number of sequences. Similarly, 92.1% of sequences belonging to *Wolbachia* were detected in only 12.4% of the samples. Finally, 8.1% of samples harbouring *Spiroplasma* contained 67% of the total number of sequences corresponding to this genus in the dataset.

Because, based on the literature, we hypothesised that *Arsenophonus* and *Wolbachia* OTUs could not be members of the tick microbiota (this hypothesis is developed in the discussion part), we also interested to their distribution in our samples. Indeed, investigating the cumulated percentages of sequences belonging to *Wolbachia* and *Arsenophonus* per sample, we observed that these 2 genera, considered together, represent on average 66.4% of sequences in the 74 infected samples and represent more than 90% of the sequences for 26 of them (data not shown).

### Temporal dynamics of the *Ixodes ricinus* microbiota

#### Principal component analysis performed on the whole dataset

**Fig. 2:**
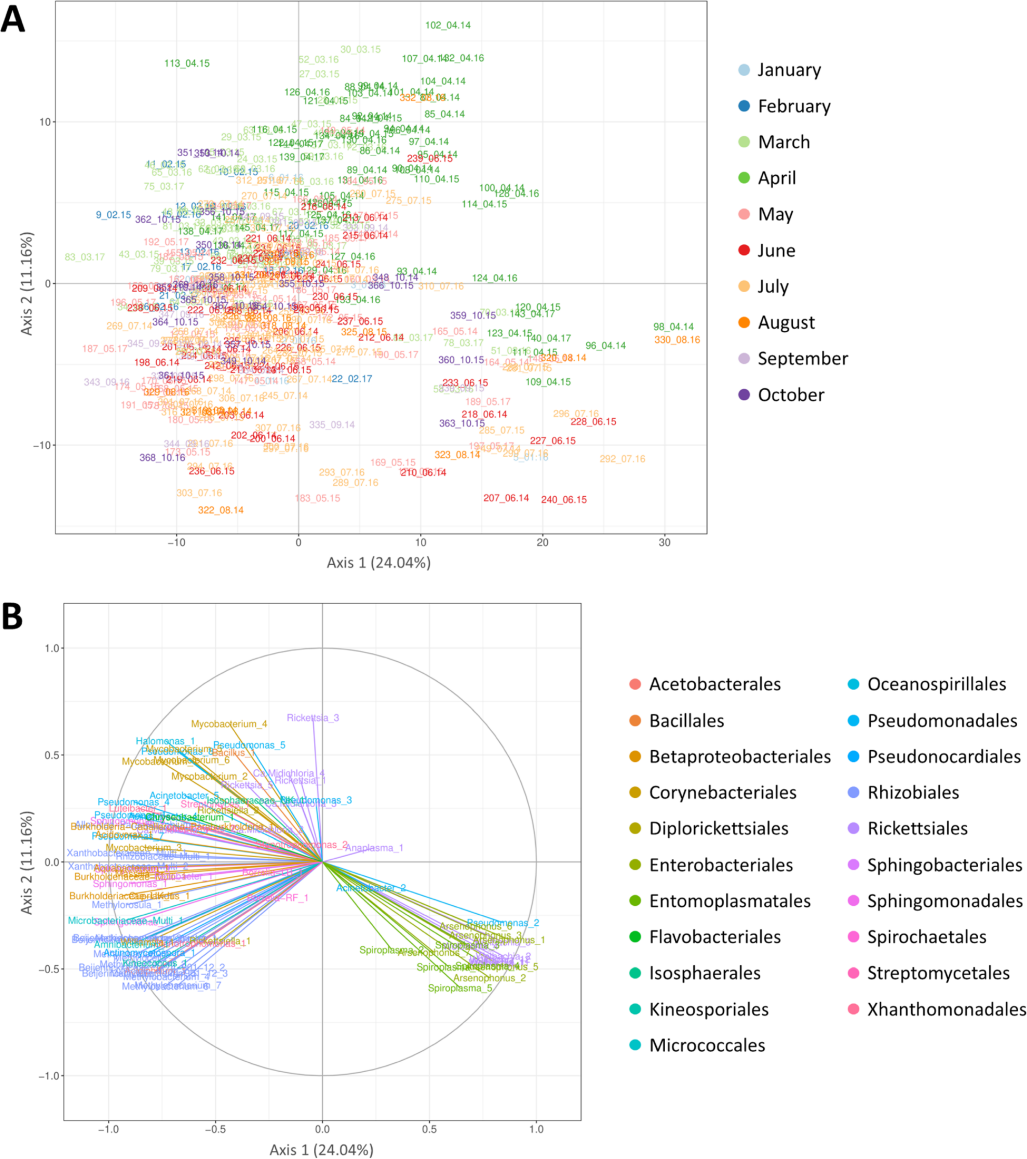
Principal component analysis performed on the whole dataset. Presented according to axes 1 (24.04%) and 2 (11.16%). **A** Sample projection of the PCA. Samples are colored according to the month of tick sampling. Plotted samples are named as following: ID_Month.Year. **B** Correlation circle of the PCA. OTUs are colored by taxonomic order

The principal component analysis was performed to determine the temporal variation of the tick microbiota, based on the relative abundances of OTUs (Fig. 2). The first two principal components PC1 and PC2 explained 24.04% and 11.16% of the total variance, respectively (Fig. 2A). All samples were clustered into three groups (deduced by graphic reading). The first one was projected in the lower right quarter and was opposed to the rest of the analysed samples, mainly according to the first axis. The small “outsider” cluster was composed of nymphs collected during different months and that seems to be randomly projected regarding the month variable. The remaining samples were divided into two clusters which were represented by ticks collected in March/April and May/June/July/August/September respectively. Note that these two clusters partially overlap. Four main genera gathering 17 OTUs seem to drive the formation of the “outsider” cluster: *Wolbachia* (OTUs 1, 2, 3, 4, 11 and 12), *Arsenophonus* (OTUs 1, 2, 3, 5, 6 and 7), *Spiroplasma* (OTUs 1, 3, 4 and 5) and *Pseudomonas* (OTU 2) (Fig. 2B). The identification of the remaining OTUs driving the two other clusters is trickier as they are not distinctly separated. In any way, it seems that OTUs belonging to the Beijerinckiaceae (Beijerinckiaceae_alphaI.cluster_1, Beijerinckiaceae_1174−901−12_1, *Methylobacterium*_1-2-3, *Methylorosula*_1, *Methylocella*_1, *Massilia*_1, *Aquabacterium*_1 and *Cupriavidus*_1), Xantobacteriaceae (Xanthobacteriaceae_Multi_1-2), Burkholderiaceae (Burkholderiaceae-UK_1, Multi_1, *Burkholderia-Caballeronia-Paraburkholderia*_1 and *Acidovorax*_1), Rhizobiaceae (Rhizobiaceae-Multi_1 and *Allo−Neo−Para−Rhizobium*_1) and Microbacteriaceae (Microbacteriaceae-Multi_1, *Amnibacterium*_1 ) families as well as OTUs *Actinomycetospora*_1, *Williamsia*_1, *Pseudomonas*_1-4-7, *Luteibacter*_4, *Sphingomonas*_1-3-4-6, *Mycobacterium*_1-3 and *Kineococcus*_1 strongly explained the variance along the negative part of the second axis.

#### Principal component analysis performed on the No Wolbachia/Arsenophonus dataset

**Fig. 3:**
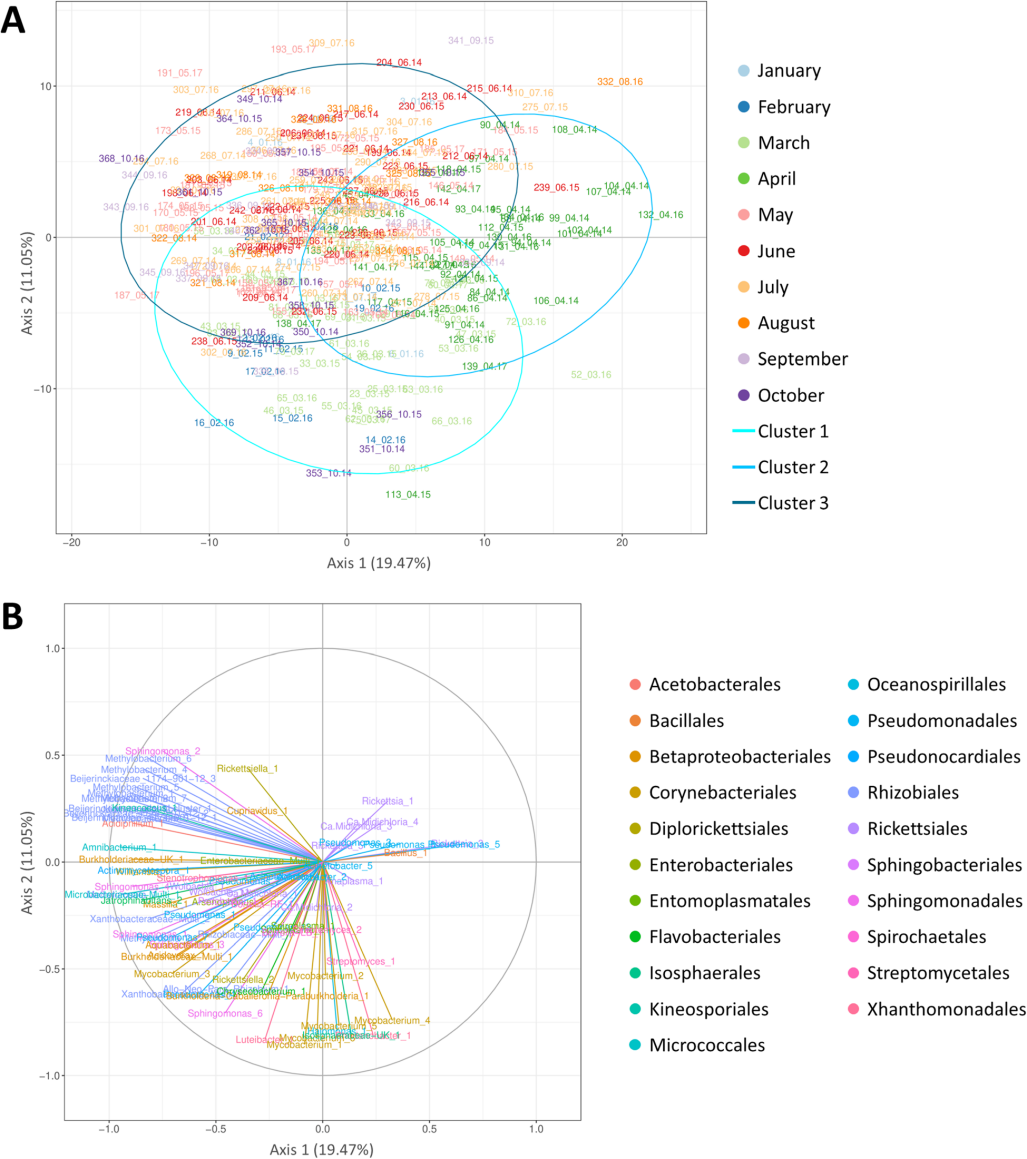
Principal component analysis performed on the dataset excluding *Wolbachia* and *Arsenophonus*-positive samples. Presented according to axes 1 (19.47%) and 2 (11.05%). **A** Sample projection of the PCA. Samples are colored according to the month of tick sampling. Plotted samples are named as following: ID_Month.Year. Cluster 1 ellipse correspond to ticks sampled in February–March, Cluster 2 ellipse correspond to ticks sampled in April and Cluster 3 ellipse correspond to ticks sampled from May to September. **B** Correlation circle of the PCA. OTUs are colored by taxonomic order

This second principal component analysis was performed to determine the temporal variation of the tick microbiota excluding the *Wolbachia-* and *Arsenophonus-*positive samples (Additional file 2) as we hypothesised that these two genera are not real members of the tick microbiota (this hypothesis is developed in the discussion part). Here, the first two principal components PC1 and PC2 explained 19.47% and 11.05% of the total variance, respectively (Fig. 3A). The clustering according to months is still visible with samples clustered into three groups (deducted by graphic reading): the cluster 1 mainly composed with samples collected in February/March, the cluster 2 with samples collected in April and the third cluster regrouping mainly samples collected in May/June/July/August/September. Samples collected in October seemed to be distributed in both clusters 1 and 3 (mainly on the left part of the plot, separated from the rest of the community *via* the first axis), while those collected in January do not seem to follow any particular distribution. Ticks collected in April (cluster 2) are distributed all along the second axis, but only on the right part of the plot, and therefore seemed to be separated from the rest of the community mainly *via* the first axis. By contrast, ticks in cluster 1, mainly collected in February/March, seemed to be distributed all along the first axis, but mainly on the bottom of the plot, and are consequently separated from the rest of the community mainly through the second axis. For the cluster 3, all samples seemed to be distributed on the top left corner of the plot, distinct from the two other clusters 1 and 2 mainly through the first and second axis, respectively.

Looking at the correlation circles (Fig. 3B), we can see that the positive part of the first axis (where are mainly distributed nymphs sampled in April), seemed to be mainly explained by *Rickettsia*_3 and *Pseudomonas*_5. To note that their effect is not as strong as those observed on the negative part of the same axis and implicating mainly OTUs belonging to the families Microbacteriaceae (Microbacteriaceae-Multi_1 and *Amibacterium*_1), Beijerinckiaceae (Beijerinckiaceae_1174−901−12_1-2-3, Beijerinckiaceae_Cluster-1, *Methylobacterium*_1-2-3-4-5-6-7, *Methylorosula*_1-2, *Methylocella*_1), Burkholderiaceae (Burkholderiaceae_UK_1) and Xhantobacteriaceae (Xhantobacteriaceae-Multi_2), as well as the OTUs *Sphingomonas*_1-4, *Williamsia*_1, *Actinomycetospora*_1, *Jatrophihabitans*_2, *Acidiphilium*-1 and *Kineococcus*_1. The negative part of the second axis (where samples of cluster 1 are mainly distributed), seemed to be mainly explained by *Mycobacterium*_1-5-6, Isosphaeraceae-UK_1, *Halomonas*_1, *Rhodanobacter*_1 and *Luteibacter*_1.

### *Ixodes ricinus* microbiota correlations

**Fig. 4:**
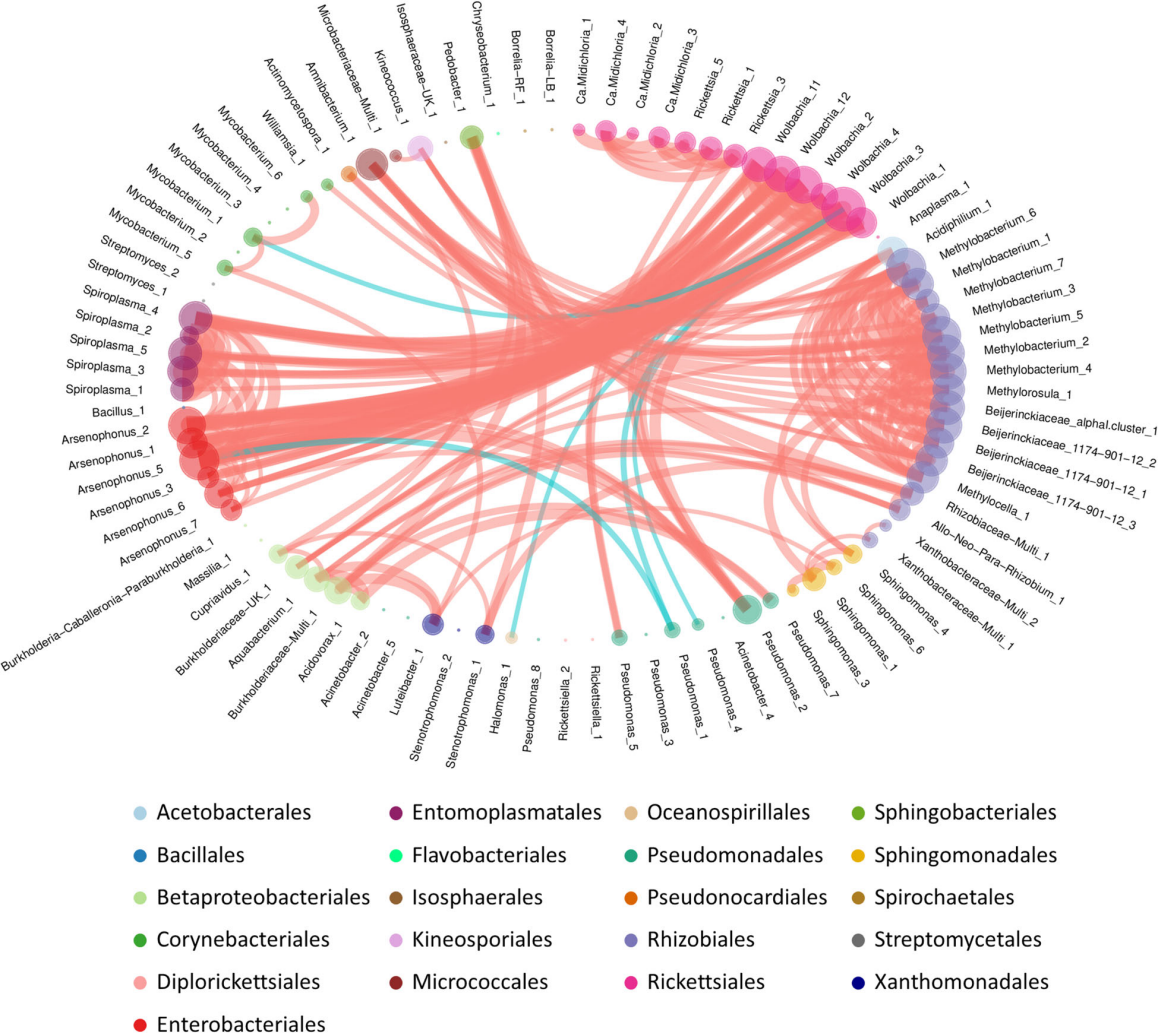
Network analysis. Representation of the significant partial correlations detected between OTUs of the whole dataset. OTU circles are colored by taxonomic order. These circles represent nodes of the networks. Their size is proportional to the sum of the incoming edge weights. Thickness of the edge is proportional to the strength of the observed partial correlation. Positive partial correlations are represented by red edges, negative partial correlations are represented by turquoise edges

Thanks to the network analysis performed on the whole dataset, we observed a total of 224 significant partial correlations between 89 OTUs. Interestingly, 97.8% of these partial correlations were positive (Fig. 4). Positive partial correlations frequently occurred between OTUs belonging to the same genus or family, as it is the case for OTUs belonging to: *Wolbachia*, *Arsenophonus*, *Spiroplasma*, *Ca*. Midichloria, *Rickettsia*, *Mycobacterium*, *Sphingomonas* and Beijerinckiaceae (including Beijerinckiaceae_1174-901-12, Beijerinckiaceae_alphal.cluster, *Methylobacterium*, *Methylorosula* and *Methylocella*). By contrast, this pattern was not observed between any of the *Pseudomonas* OTUs.

In the network, five main clusters of OTUs presenting similar connection profiles were detected (Additional file 3). Clusters 1 and 5 seem to be composed of environmental OTUs. Cluster 1 contained ten OTUs (Rhizobiaceae-Multi_1; Allo-Neo-Para-Rhizobium_1; *Pseudomonas*_7; *Stenotrophomonas*_1, *Luteibacter*_1; *Acidovorax*_1; *Burkholderiaceae*-Multi_1; *Aquabacterium*_1; *Cupriavidus*_1; *Pedobacter*_1), including five belonging to the Betaproteobacteriales while other OTUs belong to four other orders: Sphingobacteriales Xanthomonadales Rhizobiales and Pseudomonadales. Cluster 5 is composed of 17 OTUs (*Acidiphilium*_1; *Methylobacterium*_6; *Methylobacterium*_1; *Methylobacterium*_7; *Methylobacterium*_3; *Methylobacterium*_5; *Methylobacterium*_2; *Methylobacterium*_4; *Methylorosula*_1; Beijerinckiaceae_alphaI.cluster_1; Beijerinckiaceae_1174-901-12_2; Beijerinckiaceae_1174-901-12_1; Beijerinckiaceae_1174-901-12_3; *Methylocella*_1; Burkholderiaceae-UK_1; *Amnibacterium*_1; *Kineococcus*_1), mainly belonging to the Rhizobiales order (13 OTUs). Other OTUs of this cluster belong to Betaproteobacteriales, Micrococcales, Kineosporiales and Acetobacterales orders. Two other clusters are mainly composed of OTUs belonging to symbiotic or pathogenic genera (clusters 2 and 4). Cluster 2 is composed of 6 OTUs (*Ca*. Midichloria_4; *Ca*. Midichloria_3; *Rickettsia*_5; *Rickettsia*_1; *Rickettsia*_3; *Pseudomonas*_5) mainly belonging to the Rickettsiales order, and more precisely to the genera *Ca*. Midichloria and *Rickettsia*, while one of them belong to the Pseudomonadales order, more precisely to the Pseudomonas genus. Cluster 4 is composed of 17 OTUs (*Wolbachia*_11; *Wolbachia*_12; *Wolbachia*_2; *Wolbachia*_4; *Wolbachia_3*; *Wolbachia*_1; *Pseudomonas*_2; *Arsenophonus*_7; *Arsenophonus*_6; *Arsenophonus*_3; *Arsenophonus*_5; *Arsenophonus*_1; *Arsenophonus*_2; *Spiroplasma*_1; *Spiroplasma*_3; *Spiroplasma*_5; *Spiroplasma*_4), including six belonging to the *Wolbachia* genera of the Rickettsiales order, six to the *Arsenophonus* genus of the Enterobacteriales, four to the *Spiroplasma* genus of the Entomoplasmatales order and one to the *Pseudomonas* genus of the Pseudomonadales order. Cluster 3 is the largest with 39 OTUs that have in common that they have little or no correlations with other OTUs. Finally, although the following OTUs do not belong to the same clusters, negative partial correlations were observed between *Pseudomonas*_1 and both *Arsenophonus*_5 and *Wolbachia*_3. *Wolbachia*_3 was also negatively correlated to *Pseudomonas*_4, *Mycobacterium*_1 and *Halomonas*_1.

### Links between the presence of tick-borne pathogens and the *Ixodes ricinus* microbiota

#### Microbiota structure comparison between TBP-positive and TBP-negative samples

As expected, the comparison of OTU abundances between each group of TBP-positive samples (*Rickettsia*, *Borrelia* and *Anaplasma*) and TBP-negative samples allowed to detect a significantly higher abundance of OTUs belonging to the genus of the tested TBP (Additional files 4, 5, 6). Concerning *Rickettsia*-positive samples, seven other OTUs were also harbouring significantly different abundances compared to negative samples: *Ca*. Midichloria_3, *Pseudomonas*_3-5-8, *Bacillus*_1 and Rhizobiaceae-Multi_1 were significantly more abundant in *Rickettsia*-positive samples while *Spiroplasma*_1 was significantly less abundant. In *Borrelia-* and *Anaplasma*-positive samples, no other OTUs than those belonging to the corresponding tested genera were significantly over- or underrepresented.

#### Microbial network comparison between TBP-positive and TBP-negatives samples

The network analysis was performed on 5 different datasets. First on samples positive for *Rickettsia*, *Borrelia* or *Anaplasma*; then on samples negative for all these genera and finally on all the samples included in the abovementioned datasets, but considering a covariate correcting for the effect of TBP presence. As performed for the total network, OTUs presenting similar connection profiles were investigated *via* SBM. However, due to the high parsimony level of these networks, this analysis could only distinguish at best two clusters: connected samples from those presenting no correlations. Nonetheless, the determination of Kendall’s τ, integrating the edge appearance rank for all the calculated networks, allowed us to compare them and observe significant differences between all the obtained networks (Additional file 7).

**Fig. 5:**
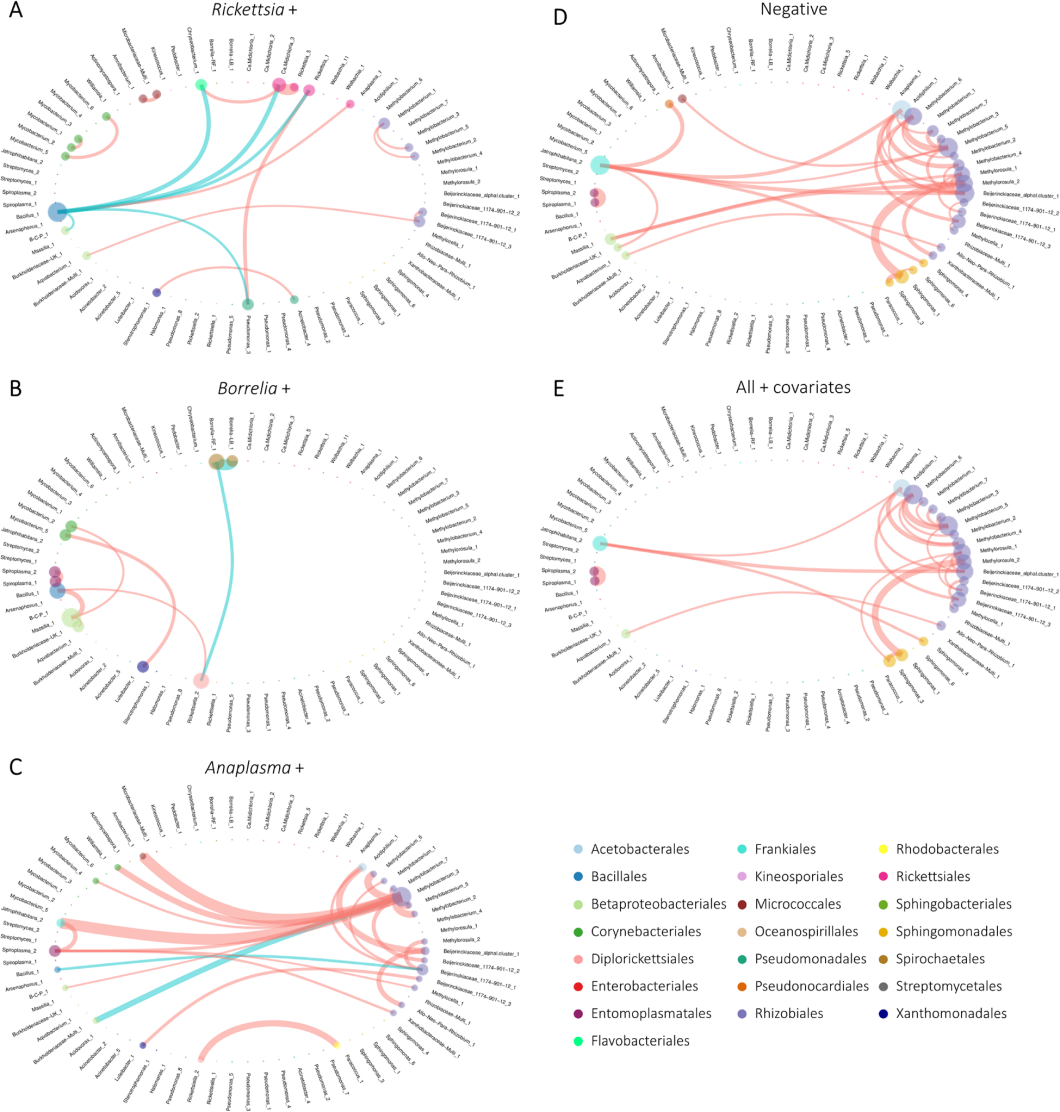
Network analysis. Representation of the significant partial correlations detected between OTUs of the TBP dataset. **A** Considering only samples positive for *Rickettsia*, **B** considering only samples positive for *Borrelia*, **C** considering only samples positive for *Anaplasma*, **D** considering only negative samples and **E** considering positive and negative samples as well as a covariate accounting for the presence of TBPs. OTU circles are colored by taxonomic order. These circles represent nodes of the networks. Their size is proportional to the sum of the incoming edge weights. Thickness of the edge is proportional to the strength of the observed partial correlation. Positive partial correlations are represented by red edges, negative partial correlations are represented by turquoise edges. For representation reasons, the OTU *Burkholderia*-*Caballeronia*-*Paraburkholderia*_1 was abbreviated to B-C-P_1

In the network analysis performed on positive ticks for *Rickettsia*, we observed 17 significant partial correlations, five were negative (Fig. 5A). Several members of the *Rickettsia* genus were positively correlated to OTUs belonging to *Ca*. Midichloria (*Rickettsia*_5/*Ca*. Midichloria_3) and *Pseudomonas* (*Rickettsia*_1/*Pseudomona*s_3) genera, as already observed in the total dataset network. *Rickettsia*_1, *Pseudomonas*_3 and *Ca*. Midichloria_3 were negatively correlated to *Bacillus*_1. This latter OTU was also negatively correlated with 2 OTUs belonging to environmental genera, *Burkholderia*-*Caballeronia*-*Paraburkholderia*_1 and *Chryseobacterium*_1 (that was also positively correlated to *Ca*. Midichloria_3), and positively correlated with *Anaplasma*_1. *Bacillus*_1 therefore appeared as a key member of this network exhibiting several partial correlations with environmental bacteria but particularly with some pathogenic and symbiotic genera. Several positive partial correlations were also observed between OTUs belonging to environmental genera, linking several OTUs within their own genus (*Methylobacterium* and *Mycobacterium*) or between several different genera or families (Beijerinckiaceae and *Aquabacterium*; *Stenotrophomonas* and *Acinetobacter* as well as Microbacteriaceae and *Amnibacterium*). We then performed a network analysis considering only ticks positive for *Borrelia* (Fig. 5B). Only 8 significant partial correlations were observed, including 2 negatives. Negative partial correlations were observed between first *Borrelia*-RF_1 (responsible of Relapsing Fever) and *Borrelia*-LB_1 (responsible of Lyme Borreliosis) and then between *Borrelia*-RF_1 and *Rickettsiella*_2 (Fig. 5B). A positive partial correlation implying *Rickettsiella*_2 and *Bacillus*_1, and another one implying *Spiroplasma*_1 and 2 were also observed as well as several positive partial correlations implying environmental genera (*Bacillus*_1, *Massilia*_1, *Aquabacterium*_1 and *Mycobacterium*_2, as well as *Mycobacterium*_5 and *Luteibacter*_1). In the network analysis performed on ticks only positive for *Anaplasma*, we observed 23 significant partial correlations with 2 identified as negative (Fig. 5C). A positive partial correlation was observed between *Rickettsiella*_2 and *Parracoccus*_1. *Spiroplasma*_2 was positively correlated with *Jatrophihabitans*_2 and several members of the Rhizobiales order (*Methylobacterium*_3, *Methylocella*_1 and *Xhantobacteraceae*_Multi_1). Other partial correlations concerned environmental bacteria, with members of the Rhizobiales order, correlated with each other or with OTUs belonging to environmental genera corresponding to other orders (Acetobacterales, Bacillales, Betaproteobacteriales, Corynebacteriales, Micrococcales and Xanthomonadales). No correlations were observed between *Anaplasma* and the other members of the bacterial community. The negative network (Fig. 5D), comprising only free pathogen samples, was composed of 33 positive partial correlations and no negative correlation. In this network, a strong partial correlation was observed between *Spiroplasma*_1 and 2. Most of the other correlations were observed between members of the Rhizobiales order, mainly between each other, but also with environmental OTUs belonging to other orders, such as Acetobacterales, Betaproteobacteriales, Frankiales, Micrococcales, Pseudonocardiales and Sphingomonadales. Although it contains less partial correlations (25), the total network corrected for TBP effects (Fig. 5E) is very similar to the negative network. Indeed, partial correlations observed between *Spiroplasma*_1 and 2, as well as most partial correlations involving Rhizobiales order between each other and with OTUs belonging to other orders are also observed in this network.

## Discussion

### *Ixodes ricinus* microbiota diversity and composition

Investigating the microbiota of 371 *I. ricinus* nymphs, we identified 109 bacterial genera, i.e. within the range of previous observations in this tick species [6, 12]. Similarly, the mean Shannon diversity index (=2.1) [22] is in the range reported in the literature for *Ixodes* ticks [8, 20, 40, 41]. However, these values are known to fluctuate, mainly according to the tick instars, species or localization, and are therefore difficult to compare [5, 8, 10, 12, 17, 41–45]. Furthermore, not all these studies used negative controls to identify potential contaminating OTUs and remove them from their datasets, thus calling for caution when attempting to draw conclusions concerning these differences, particularly knowing that such OTUs can represent more than 50% of the sequences detected in tick samples [22]. In our dataset, three quarters of the sequences belonged to five genera: *Arsenophonus*, *Ca*. Midichloria, *Rickettsia*, *Wolbachia*, *Spiroplasma*, all of which corresponded to well-known maternally inherited bacteria in arthropods [46]. Their high prevalence in the dataset suggest they are widely distributed in a large proportion of tick samples and was clearly the case in *Ca*. Midichloria, in which most of the detected sequences were found in half the tick samples. This result is in line with previous reports that almost 100% of *I. ricinus* females and larvae, and almost 50% of nymphs and males were infected by this endosymbiont [47–50]. The four other genera (*Rickettsia*, *Spiroplasma*, *Wolbachia* and *Arsenophonus*) were mainly detected in less than 17% of samples (7.8%, 8.1%, 12.4% and 16.7%, respectively). The contrast between the high proportions of sequences corresponding to these genera and the small number of samples infected is due to their high relative abundance in infected samples. Focusing on *Rickettsia*, the sequences corresponded to *R. helvetica*, which have already been detected in these samples [26] and known to exhibit a high bacterial load in ticks [51, 52]. Concerning *Arsenophonus* and *Wolbachia*, while the high number of sequences detected in the dataset as well as their high cumulated percentage in infected samples suggest they are dominant members of the *I. ricinus* microbiota, their detection could be due to the presence of the parasitoid wasp, *Ixodiphagus hookeri*, in ticks [53, 54]. This high prevalence, almost excluding endogenous tick bacteria, prompted us to wonder about the potential role of this symbiont in the colonisation of *I. ricinus* by *I. hookeri*. Indeed, as previously hypothesised by Plantard et al. [53], the presence of *Wolbachia* could induce an immune reaction in the tick that could have a negative impact on other microorganisms hosted by the tick. This kind of strategy, dealing with the host immune system, could allow the symbiont proliferation and so the egg maintenance, as it has already been observed in *Drosophila stimulans*, in which the presence of *Wolbachia* reduced the ability of the host immune system to encapsulate the eggs of the parasitoid wasp *Leptopilina heterotoma* [55]. Finally, among the most frequently detected genera in the *I. ricinus* microbiota, *Spiroplasma* is a maternally inherited symbiont particularly well known in arthropods, including ticks [56]. With most of their sequences detected in only 8% of tick samples, this bacterial genus probably represents secondary symbionts [56], which are not essential for the survival of their host but may confer conditional adaptive advantages, such as protecting their host against pathogens and natural enemies [57, 58]. This is probably the case for *I. ricinus* ticks potentially infected with the parasitoid wasp *Ixodiphagus hookeri*, as we discuss later in this section. Among the remaining genera, several are known to circulate in *I. ricinus* ticks, including *Rickettsiella* (a maternally inherited bacterium in arthropods), *Borrelia* (Lyme Borreliosis and Relapsing Fever) and *Anaplasma*. Some others, including *Methylobacterium*, *Mycobacterium*, *Pseudomonas*, *Stenotrophomonas*, *Williamsia*, *Bacillus*, *Chryseobacterium*, *Acinetobacter* and *Allorhizobium-Neorhizobium-Pararhizobium-Rhizobium*, as well as multi-affiliated OTUs belonging to Rhizobiaceae and Microbacteriaceae, which have already been detected in *Ixodes ricinus* ticks [6, 12, 16, 17, 19], and have also been identified in the environment [59–64]. While very few of the abovementioned references reported performing negative controls while studying tick microbiota [12, 16], it is important to keep in mind that some of these genera could also be contaminants that arise during the extraction or amplification steps [22, 30, 65–67]. Our dataset was subject to a thorough contaminant filtering, based on negative control composition [22], thereby reducing the risk that these OTUs correspond to contaminants. Nonetheless, the question remains as to whether these environmental taxa belong to the internal tick microbiota or to the cuticular microbiome? Probably to both. Indeed, Binetruy et al. [68] demonstrated that surface sterilisation of the tick using ethanol (which we used in the present study) was not as efficient as bleach at removing bacterial DNA from the tick cuticle. These authors also showed that environmental taxa, only detected in ethanol cleaned whole ticks, belonged to the same family as those directly detected on the tick cuticle by swabbing. However, they also found some of these taxa in the gut of ticks. While studying the microbiota in organs of *I. ricinus* ticks previously cleaned with bleach, Hernández-Jarguín [19] also detected bacterial genera commonly found in the environment.

### Temporal variability of *Ixodes ricinus* microbiota

We first performed a principal component analysis of the whole dataset to characterise the temporal dynamics of the *I. ricinus* microbiota. Our results provide evidence that the *I. ricinus* microbial communities vary markedly, with two main clusters, the first grouping the microbial communities identified in March–April and the second grouping the microbial communities identified from May to September. Interestingly, this temporal pattern was observed for samples belonging to each of the 3 years, suggesting predictable temporal differences in the *I. ricinus* microbial community structure. A third cluster was also identified and corresponded to nymphs collected across the 3 years. This “outsider” cluster was mainly driven by members of the genera *Wolbachia*, *Arsenophonus* and *Spiroplasma*. As mentioned above, the presence of *Wolbachia* and *Arsenophonus* species in *I. ricinus* ticks is probably due to the presence of the parasitoid wasps, *Ixodiphagus hookeri* [53, 54], which depends entirely on the tick for its development. In their study, Plantard et al. [53] reported that almost 100% of *I. hookeri* were infested by *Wolbachia pipientis.* These authors also showed that all unfed tick nymphs parasitised by *I. hookeri* also harboured *Wolbachia*, while (with only one exception) non-infected ticks were *Wolbachia*-free. *Wolbachia* was shown to be vertically transmitted from the female *I. hookeri* to its eggs and the subsequent generation harboured *Wolbachia*. Similarly, Bohacsova et al. [54] detected the symbiont *Arsenophonus nasoniae* only in nymphs infected by the wasp *I. hookeri*, and almost 30% of the wasp population was infested by *A. nasoniae*. In this context, through the study of tick microbial communities, we suggest that identifying the temporal dynamics of both *Wolbachia* and *Arsenophonus* could serve as a proxy to characterise both the infection rate and temporal dynamics of *I. hookeri* in *I. ricinus*, even if it would probably be easier and more efficient to use primers or probes that specifically match the parasitoid DNA. While the presence of *Wolbachia* and *Arsenophonus* is thus probably linked to the presence of *I. hookeri* in ticks, we hypothesised that their presence in our dataset could alter our characterisation of the temporal dynamics of the *I. ricinus* microbial communities. We therefore chose to perform another PCA analysis after removing all samples harbouring one or both genera *Wolbachia* and *Arsenophonus*. As expected, the new PCA performed on the “reduced” dataset improved separation of the clusters. We finally distinguished three main clusters. Whatever the sampling year, this analysis mainly distinguished ticks collected in February/March (cluster 1), from those sampled in April (cluster 2) or from those sampled from May to September (cluster 3), with ticks sampled in October distributed in both clusters 1 and 3, suggesting that the factors which drove the tick microbial communities were probably the same in the three consecutive years. Interestingly, we also observed that the OTUs that best explained the dynamics of the tick microbial community structure were neither tick symbionts nor pathogens but were genera or families that are characteristic of the environment (i.e. *Sphingomonas*, *Williamsia*, *Actinomycetospora*, *Jatrophihabitans*, *Acidiphilium*, *Kineococcus*, *Mycobacterium*, *Halomonas*, Microbacteriaceae, Beijerinckiaceae, Xhantobacteriaceae, Burkholderiaceae, Rhodanobacteraceae and Isosphareaceae). Considering that these OTUs belong to the cuticular microbiome (as discussed above), the fluctuations we observed could therefore correspond to variations in the environmental microbiota [69]. However, these OTUs could also correspond to bacteria acquired by ticks from the environment during water uptake for example, or through the tick’s other orifices such as spiracles and the anal port. This was suggested by Narasimhan and Fikrig [70] who observed that ticks hatched in a sterile environment harboured a significantly different gut microbiota than those hatched in “normal” conditions [71]. We hypothesise that the temporal variations we observed in our analysis could thus also be due to differences in the composition of the environmental microbiota. Otherwise, abiotic factors such as temperature, which are likely to follow the same yearly pattern, have been shown to influence the diversity of the microbiota of *I. scapularis* ticks [45]. Furthermore, blood meal and host identity have also been shown to influence the diversity of tick microbiota [11, 40, 71, 72]. Finally, considering the life cycle of ticks, one other hypothesis which could explain this pattern is that tick microbiota structure and composition could be linked to the feeding status of ticks. While quantifying the lipid content of *Ixodes ricinus* ticks sampled monthly over a period of several years, Randolph et al. [73] and Abdullah et al. [74] managed to discriminate tick feeding cohorts questing at different periods of the year. Both studies reported that lipid reserves were higher in ticks collected at the end of the year than in those sampled from the end of spring and throughout summer. According to Abdullah et al. [74], ticks sampled at the end of the year (high lipid reserves), correspond to those that succeeded in obtaining their blood meal in the previous spring and emerged in autumn. The ticks sampled after the end of the year and at the beginning of the following spring, presenting a relatively high lipidic reserves that trend to increase towards March, were hypothesised to correspond to a mix of tick populations: (1) those just emerging, with high lipid reserves and (2) those that already emerged in autumn, with lower lipid levels. Ticks still questing at the end of spring or during summer were hypothesised to correspond to those that failed to find a host and had almost exhausted their lipid reserves. In this case, one could hypothesise that the ticks in cluster 3 (mainly ticks sampled from May to September) correspond to those with very few energy reserves, while ticks in cluster 1 (sampled in February/March) correspond to those with higher energy reserves. Ticks sampled in October, distributed in clusters 1 and 3, could correspond to a mix of two populations: (1) those with high lipid reserves (i.e. which had fed the previous spring and had just emerged) and (2) those whose energy reserves were almost exhausted (which had fed the previous year), as observed for nymphs sampled in October 2015 and 2016 in Abdullah et al. [74]. However, this hypothesis does not elucidate the clustering of the ticks sampled in April, thus suggesting that other factors (related to the life cycle of ticks or their environment), could be at the origin of this pattern. One can further hypothesise that the community patterns of bacteria are also related to physiological stress of the tick, the microbiota “supporting” the tick’s physiological requirements, perhaps in agreement with differences in nutritional stress, as suggested above, or perhaps to abiotic stress linked to temperature or saturation deficit. In any case, further studies will be necessary to elucidate this point and confirm or invalidate these hypotheses.

### *Ixodes ricinus* microbial community interactions

Identifying and understanding tick-borne microbe interactions is a precondition for the development of new strategies to control ticks and tick-borne diseases using the tick microbiome. Using partial correlation networks, we assessed which tick bacterial OTUs were correlated in order to identify possible associations between tick microbial community members.

First, used on the whole dataset, network analysis revealed that more than 97% of detected interactions between members of the *I. ricinus* microbial community were positive. Taxa with positive associations have usually been interpreted as functional guilds of organisms performing similar or complementary functions [75, 76] or featuring interactions shaped by interspecies cross-feeding [77], although sometimes they may mainly reflect shared habitat preferences [78]. Similarly, negative associations may reflect interactions including competition and niche partitioning. The vast majority of positive correlations observed therefore suggest that tick microbial communities favour mutualism and perform similar or complementary functions. But let us keep in mind that the correlations observed in our study were obtained by examining entire individuals and may impair the observation of associations at a finer scale (e.g. organs) [27].

Due to the low infection rate observed in the nymphs studied [26], the low proportion of ticks in our dataset that tested positive for pathogens could bias the analyses and conceal important information such as crucial interactions between TBPs and members of the tick microbiota. To overcome this bias, we decided to perform supplemental analysis to compare TBP-positive and TBP-negative samples. Further, to get rid of effects that could hamper or skew the observation of the impact of TBPs, samples that appeared to be parasitised by *I. hookerii* (i.e. with numerous sequences belonging to *Arsenophonus* and/or *Wolbachia*) were excluded from this analysis, and a covariate correcting for the effect of the sampling season was added, compared to the analysis performed on the whole dataset. The resulting negative network was mainly composed of partial correlations linking environmental OTUs, and very few correlations remained compared to the network obtained using the whole dataset. These results suggest that most correlations previously observed in the total network are linked to the presence of TBPs, the response to the parasitoid or the seasonal effect. This further strengthens the importance of these variables in the tick microbial community structure and underlines the adaptability of the tick microbiota to variable conditions. Comparing negative samples with those infected by pathogens enabled us to demonstrate that with TBPs, the structure and correlations of tick microbial communities were considerably modified in presence of TBPs and according to the concerned pathogen. Indeed, in ticks infected by *Rickettsia*, the proportions of several OTUs (i.e. *Spiroplasma* symbiont and environmental OTUs) were significantly affected. Moreover, more negative correlations were detected in TBP samples, suggesting much more competition between members of the tick microbial communities in presence of TBPs. In addition, in the “free pathogen” network, we observed a lot of correlations involving environmental OTUs (i.e. belonging to Rhizobiales, Acetobacterales, Betaproteobacteriales, Frankiales, Micrococcales, Pseudonocardiales and Sphingomonadales orders), whereas these correlations decreased or even disappeared in infected ticks, suggesting that the presence of TBPs affects these microbial interactions. Two main hypotheses emerged from these results: the tick microbiota are initially disturbed and thus favour infection by pathogens, or the presence of pathogens affects the structure of the tick microbiota. Because the structure and interactions of tick microbial communities are completely different depending on the pathogen concerned, we first suggest that the presence of pathogens in ticks is likely to affect the other members of the tick microbiota. However, disturbance of the microbiota could facilitate the installation of pathogens in ticks. To give an example, the presence of *Anaplasma phagocytophilum* in *I. scapularis* could modify the gut microbiota whose modification could, in turn, disrupt the integrity of the gut membrane and facilitate entry by the pathogen [79]. Based on our data, we are currently unable to conclude and further experiments are required to this end.

### Interactions between non-pathogenic OTUs

First, we detected in the a cluster of OTUs (cluster 4) grouping several OTUs belonging to *Wolbachia*, *Arsenophonus* and *Spiroplasma* OTUs and one *Pseudomonas* OTU (*Pseudomonas*_2), positively linked to each other in the whole network. As previously mentioned, detecting *Wolbachia* and *Arsenophonus* in *I. ricinus* is probably evidence for the presence of the parasitoid *I. hookeri* in these ticks [53, 54]. While the presence of the deer-associated *Anaplasma phagocytophilum* in ticks has been previously shown to be positively correlated with the presence of *I. hookeri*, due to the way of life/mode of hunting of this parasitoid [80], we found no correlation between this pathogen and *Wolbachia* or *Arsenophonus* OTUs in the present study. Conversely, *Spiroplasma*, identified as arthropod symbionts including ticks [46, 56, 81–84], were closely linked to the dynamics of these two genera. Identified as a “male killer” in many other arthropods [85–90], our results on nymphs alone do not allow us to conclude on this potential role. Nonetheless, assessing the proportions of contaminant sequences when characterising both nymph and adult tick microbial communities with high-throughput sequencing, we detected a high proportion of *Spiroplasma* sequences in males [22], suggesting that these *Spiroplasma* species do not cause specific male mortality in *I. ricinus* ticks. Furthermore, it should be noted that no evidence of sex-ratio distortion was found by Binetruy et al. in the population of *Rhipicephalus decoloratus* ticks infected with *Spiroplasma ixodetis* [56]. Assuming that the detection of both *Wolbachia* and *Arsenophonus* is probably due to the infection of ticks by *I. hookeri*, we hypothesise that *Spiroplasma* is upregulated by the presence of the parasitoid in ticks and may be a defensive response mechanism against *I. hookeri*, as previously reported in *Drosophila melanogaster* infested by two species of parasitoid wasps [91]. Nevertheless, we cannot rule out the hypothesis that, while identified in a large variety of tick species [56], *Spiroplasma* may correspond to a symbiont of *I. hookeri*. Besides, while *Pseudomonas*_2 was identified in this cluster and was positively linked to its other members, it should be noted that two other *Pseudomonas* OTUs (*Pseudomonas*_1 and 4) were negatively correlated with several *Wolbachia* and *Arsenophonus* OTUs. In our dataset, very few OTUs belonging to the same genus displayed contrasting patterns of correlation, but *Pseudomonas* is an exceptionally versatile genus including plant pathogens, human and animal opportunistic pathogens, and saprophytic bacteria found in water and soil exhibiting great adaptation to their environment [92]. Notably, some members of this genus have been reported to influence arthropod survival. For example, *P. fluorescens* strains have been reported to confer better survival to the *Varroa destructor* mite and the *Galleria mellonella* waxworm in presence of a fungal pathogen of *Beauveria bassiana* arthropods [93, 94]. Conversely, *P. entomophila* has been reported to be entomopathogenic for *Drosophila* [95, 96]. Such contrasted behaviour in arthropods could explain the contrasting correlations observed with *Pseudomonas* OTUs, and suggest that, like *Spiroplasma* OTUs, some of them may be involved in defense mechanisms against *I. hookerii*.

Two other groups of OTUs, comprising non-pathogenic OTUs, were identified in the total network, and correlations involving several members of these clusters were still observed in the negative and covariate corrected networks. These were the OTUs belonging to clusters 1 and 5 that belong to the Betaproteobacteriales, Sphingobacteriales, Xanthomonadales, Rhizobiales and Pseudomonadales orders in the case of cluster 1, and of OTUs belonging to Rhizobiales, Betaproteobacteriales, Micrococcales, Kineosporiales and Acetobacterales orders in the case of cluster 5. All these OTUs belong to genera and families of microorganisms commonly found in the environment [62, 97–102], and known to potentially infect ticks. The high connectivity displayed by these OTUs may reflect similar functions between OTUs adapted to contrasting environmental conditions (functional redundancy). However, such a scenario would have probably resulted in many more negative correlations between these OTUs. On the other hand, these findings may indicate reduced functional capabilities of each OTU and hence a complementary functional role for these taxa in ticks. All these hypotheses need to be investigated in future works on the functional ecology of tick microbiota.

Finally, two correlations linking OTUs belonging to the symbiotic genus *Rickettsiella* to OTUs belonging to the environmental genera *Paracoccus* and *Bacillus* were observed in the *Anaplasma* and *Borrelia* networks, respectively. While the *Rickettsiella* genus has been detected in several species of ticks [46], notably in *I. ricinus* ticks [12, 17–19, 46], little is known about the importance of this maternally inherited bacterium in tick species. Its correlations with environmental OTUs in the presence of TBPs could mean it plays a particular role in the pathogenic context in *Ixodes ricinus* ticks.

### Interactions between tick-borne microbiota and pathogens

Interestingly, we found correlations between *Ca*. Midichloria and *Rickettsia* OTUs that were strong enough to be observed not only in the *Rickettsia* network, but also in the whole dataset network where these OTUs were grouped in cluster 2. While our sequencing approach does not allow us to identify *Rickettsia* at the species level, we hypothesise that they correspond to pathogenic agents, as *Rickettsia helvetica* was specifically detected in these same samples [26]. In addition, *Ca*. Midichloria abundance was significantly higher in *Rickettsia*-positive samples than in the negative ones. Considering the strong prevalence of *Ca*. Midichloria in *I. ricinus* ticks, the positive relationship between members of this genus and members of *Rickettsia* genus suggests a facilitating role for *Ca*. Midichloria in *I. ricinus* colonisation by *Rickettsia*. This hypothesis is in accordance with the results obtained by Budachetri et al. [28] who observed a positive correlation between *Ca*. Midichloria *mitochondrii* load and *Rickettsia parkeri* presence in the tick *Amblyomma maculatum*. The fact that *Rickettsia* OTUs were positively correlated with maternally inherited bacteria is particularly surprising, especially because members of this genus have been frequently reported to be involved in antagonist relationships with symbiotic or pathogenic genera [9, 10, 16, 103–107]. The potential complementarity of these two genera should be examined in more detail in the future by characterising the bacterial transcriptome or metabolome of ticks infected with these bacteria alone or in association. Interestingly, the OTU of *Borrelia* Relapsing fever (*Borrelia* RF) was negatively correlated with *Rickettsiella* and *Borrelia* Lyme Borreliosis OTUs (*Borrelia* LB). The negative correlations between both *Borrelia* groups (relapsing fever *vs* Lyme borreliosis) we identified using the 16S rDNA are in full agreement with previous results we obtained using the same tick samples and using a high-throughput microfluidic real-time PCR with specific primers and probes to detect specifically tick-borne pathogens [26]. Even though this association was not significantly underrepresented compared to a random distribution, our previous results highlighted higher prevalences of *Borrelia burgdorferi s.l.* when *B. miyamotoi* prevalences were low, and vice versa. Competition for the same niche may explain the negative correlations observed between these two groups of *Borrelia*. However, these findings contrast with those reported by Aivelo et al. [16] as these authors found positive correlations between the relapsing fever spirochete *B. miyamotoi* with *Rickettsiella* and two *Borrelia* species belonging to the group *Borrelia* Lyme borreliosis (*B. garinii, B. afzelii*). Because clinical co-infections with several TBPs are commonly reported [108–110] and are known to affect both disease symptoms and severity [111, 112], it is now crucial to identify the conditions in which *Borrelia* RF and *Borrelia* LB are in competition, or on the contrary, in which they could collaborate and thus co-infect ticks. Furthermore, and contrary to what it has been observed on *Ixodes scapularis* in the literature [113, 114], we did not detect significant differences in the structure of the tick microbiota in *Borrelia-*positive samples compared to the negative ones, with no OTUs under- or overrepresented in these samples. The difference of tick species investigated could explain such contrasting results, especially since most of the genera overrepresented in the Brinkerhoff study [114] were not detected in our study. Furthermore, Chauhan et al. [113] focused their analysis on *Borrelia burgdorferi* s.l. investigated in females, while we investigated *Borrelia* (including Lyme borreliosis and relapsing fever *Borrelia*)-positive nymphs that could be another explanation for such differences of observation in our study. Otherwise, we also observed several partial correlations between pathogens and OTUs usually known to be “environmental” bacteria. This was notably the case of a *Pseudomonas* OTU positively correlated with *Rickettsia*. In addition, this OTU and two others belonging to the same genus were more abundant in *Rickettsia-*positive samples. While several *Pseudomonas* OTUs were previously identified as contaminants and removed from the dataset analysed [22], those remaining are involved in several interactions with different members of the tick microbial community, such as *Wolbachia* and *Arsenophonus*, as already discussed above, but also with TBPs, demonstrating the versatility of members of this genus and their importance in the tick microbiota structure. *Bacillus* also appears to be a key member of the *Ixodes ricinus* microbial community linked to the presence of TBPs. While Adegoke et al*.* [115] observed higher abundances of this bacterial genus when the parasite *Theileria* was present in the tick *Rhipicephalus microplus*, our findings demonstrate a positive correlation between *Bacillus* and *Anaplasma* and a negative one with *Rickettsia*. The latter, implying *Bacillus* and *Rickettsia*, suggest potential competition between these bacteria and may be a first step to develop a future tool to control tick infections by *Rickettsia*. Furthermore, because the dynamics of environmental bacteria found in ticks varies over the year, probably due to contrasting environmental conditions, the vegetation and the tick hosts, their positive or negative interactions with pathogens suggest that their presence or absence is an important factor to take into account to better understand the temporal dynamics of TBPs. Finally, all these correlations involving “environmental” OTUs suggest that their detection in tick microbiota is probably not only the result of accidental ingestion, but more likely reflects their true adaptation within the tick microbial community.

## Conclusion

Here we reported the identification of the *Ixodes ricinus* microbiota in nymphs collected monthly in three consecutive years. These results allowed us to show that (1) the *Ixodes ricinus* microbiota is not stable over time but displays a recurrent temporal pattern that is mainly explained by the dynamics of environmental taxa; (2) the presence of TBPs is likely to disturb tick microbial community structure and hence tick/microbe interactions; (3) some specific symbionts and “environmental” bacteria may play a key role in the presence and the dynamics of *I. ricinus*-borne pathogens and in the defense against parasitoid species. While the microbial correlations identified in this ecosystem study need to be confirmed in the near future using experimental approaches, our new findings suggest that a large part of the tick microbiome, including environmental taxa, could play a role in the infectious risk associated with *Ixodes ricinus*, either through bacteria-bacteria interactions or interactions with the tick and its natural enemies. The tick microbiome diversity would thus be considered as a promising resource for the development of new controls strategies against tick and tick-borne diseases.

## Supplementary Information

**Additional file 1.** Distinction of contaminants OTUs, removed from the dataset, from genuine members of tick microbiota.

**Additional file 2 **Graphic representation of samples presenting a significant number of sequences belonging to *Arsenophonus* and *Wolbachia* genera. This figure is adapted from the principal component analysis performed on the whole dataset, presented according to axes 1 (24.04%) and 2 (11.16%). (A) Sample projection of the PCA. Samples are colored according to the month of tick sampling. Plotted samples are named as following: ID_Month.Year. Samples presented in larger format and darker colour are those that have been removed from the dataset for the subsequent analyses. (B) Correlation circle of the PCA. OTUs are colored by taxonomic order. OTUs presented in larger format and darker colour are those belonging to *Arsenophonus* and *Wolbachia* genera.

**Additional file 3.** OTUs clusters identified from the total network. OTUs presenting similar connection profiles were identified and assigned to different clusters according to a stochastic block model obtained from the corresponding binary adjacency matrix, obtained from the network inferred by PLN network, which places an edge for each non-null partial correlation (whether positive or negative).

**Additional file 4 **Microbiota structure comparison between *Rickettsia* positive and negative samples. Differential analysis comparing OTUs abundances between *Rickettsia* positive and negative samples. Differentially abundant OTUs were defined as those with a p-values < 0.05 after adjustment for multiple testing using the Bonferroni procedure.

**Additional file 5 **Microbiota structure comparison between *Borrelia* positive and negative samples. Differential analysis comparing OTUs abundances between *Borrelia* positive and negative samples. Differentially abundant OTUs were defined as those with a p-values < 0.05 after adjustment for multiple testing using the Bonferroni procedure.

**Additional file 6 **Microbiota structure comparison between *Anaplasma* positive and negative samples. Differential analysis comparing OTUs abundances between *Anaplasma* positive and negative samples. Differentially abundant OTUs were defined as those with a p-values < 0.05 after adjustment for multiple testing using the Bonferroni procedure.

**Additional file 7 **TBPs positive and negative network comparison. The different families of network generated in this study (negative, *Rickettsia*, *Borrelia*, *Anaplasma* and total corrected for TBPs effect) were compared by calculating a weighted version of the Kendall’s τ, integrating the edge appearance rank within families of networks (negative, *Rickettsia*, *Borrelia*, *Anaplasma* and total corrected for TBPs effects) as well as associated Pvalues.

## Data Availability

The datasets used for this study can be found in the European Nucleotide Archive. Project accession number: PRJEB36903 (ERP120162) – Sample accession numbers: ERS4353953-ERS4354625 – Run accession numbers: ERR3956669-ERR3957340.

## Abbreviations

*I. ricinus*: *Ixodes ricinus*
PCA: Principal component analysis
OTU: Operational taxonomic unit
TBP: Tick-borne pathogens
