# Supplementary figures and images for: Temporal patterns in Ixodes ricinus microbial communities: an insight into tick-borne microbe interactions
Source: Microbiome. 2021 Jul 3;9:153. doi: 10.1186/s40168-021-01051-8 (PMC8254910; doi:10.1186/s40168-021-01051-8)

**A**

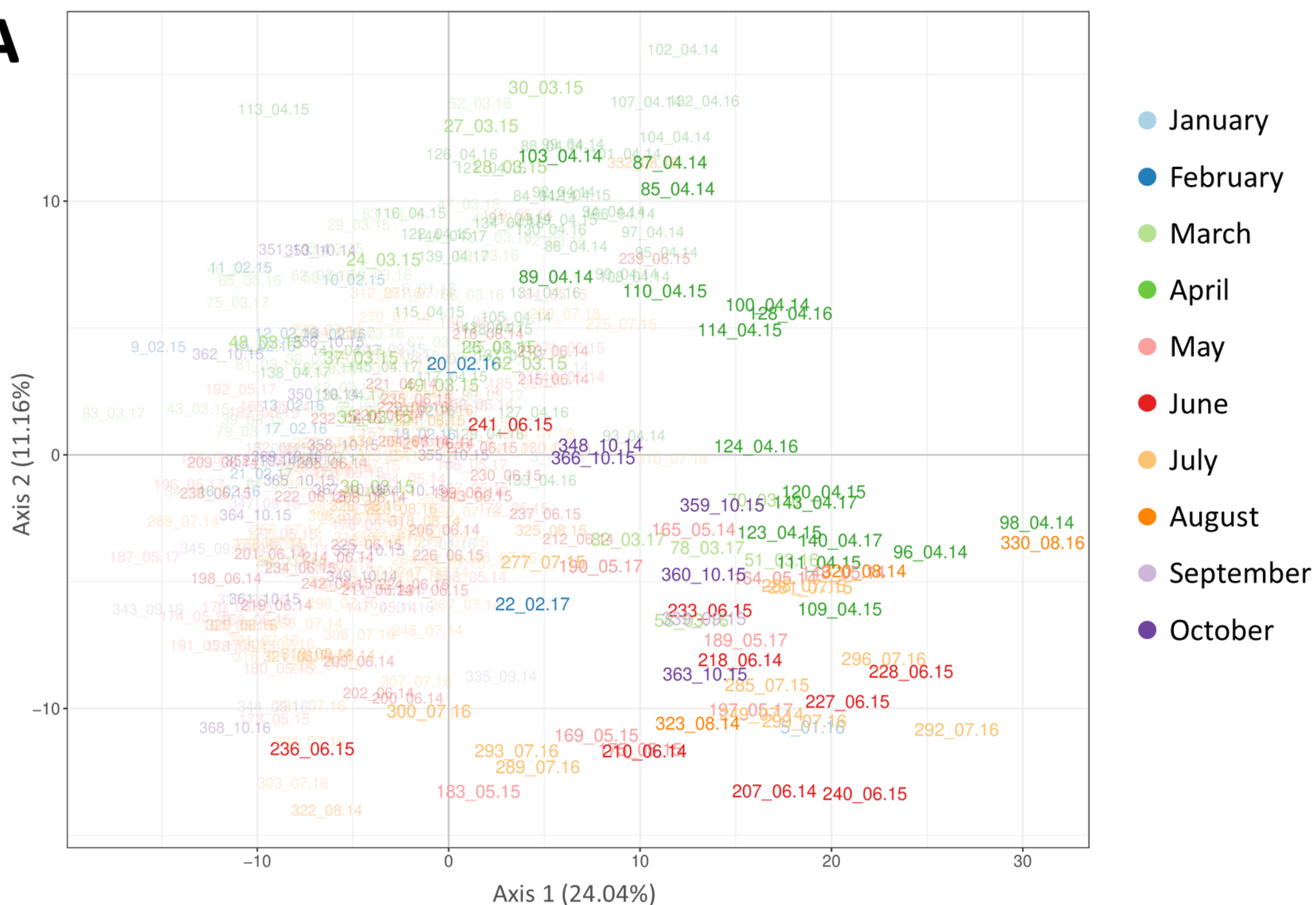

**B**

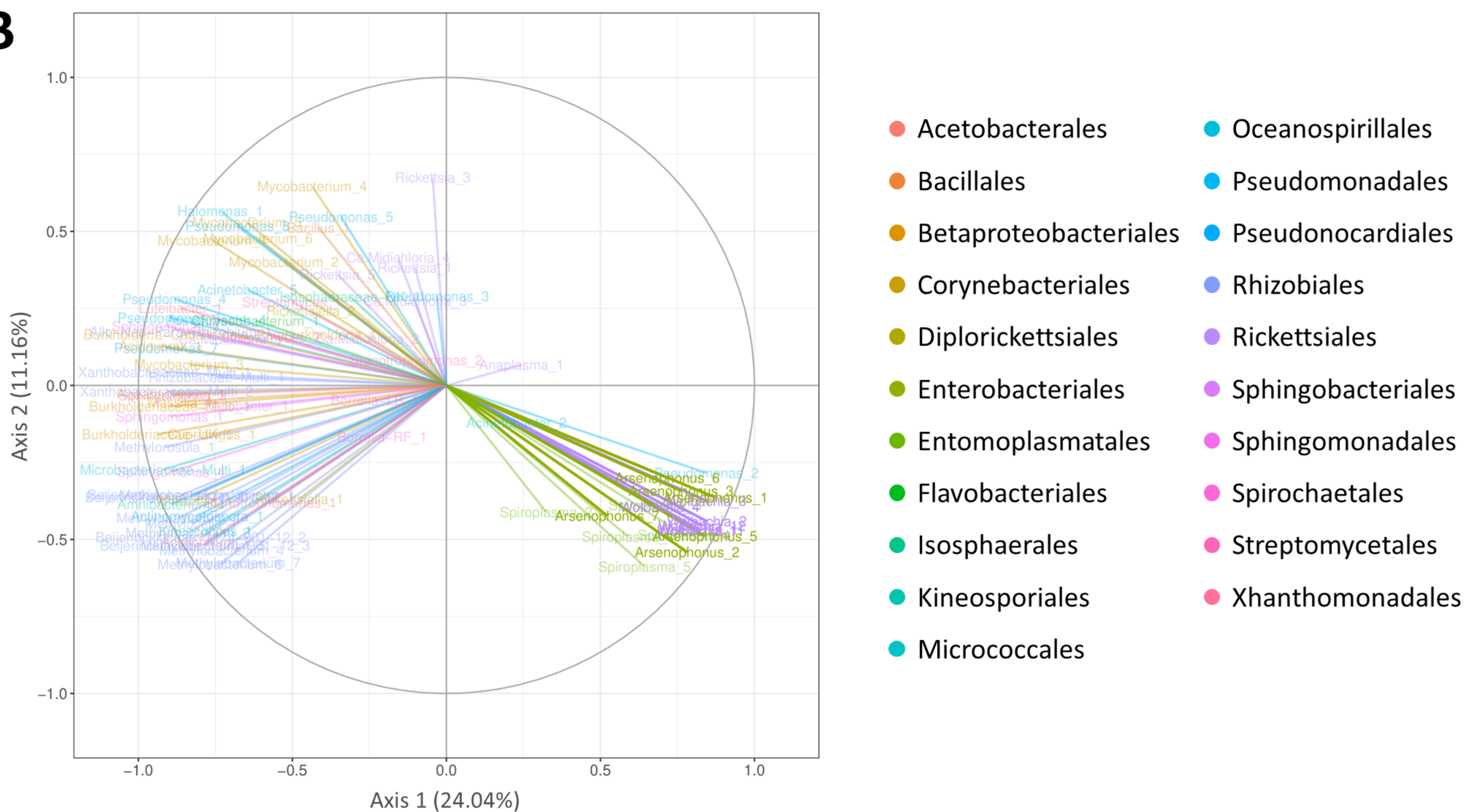

Supplement: Supplementary file 3 — Additional file 2 Graphic representation of samples presenting a significant number of sequences belonging to Arsenophonus and Wolbachia genera. This figure is adapted from the principal component analysis performed on the whole dataset, presented according to axes 1 (24.04%) and 2 (11.16%). (A) Sample projection of the PCA. Samples are colored according to the month of tick sampling. Plotted samples are named as following: ID_Month.Year. Samples presented in larger format and darker colour are those that have been removed from the dataset for the subsequent analyses. (B) Correlation circle of the PCA. OTUs are colored by taxonomic order. OTUs presented in larger format and darker colour are those belonging to Arsenophonus and Wolbachia genera. [file 40168_2021_1051_MOESM3_ESM.pdf]

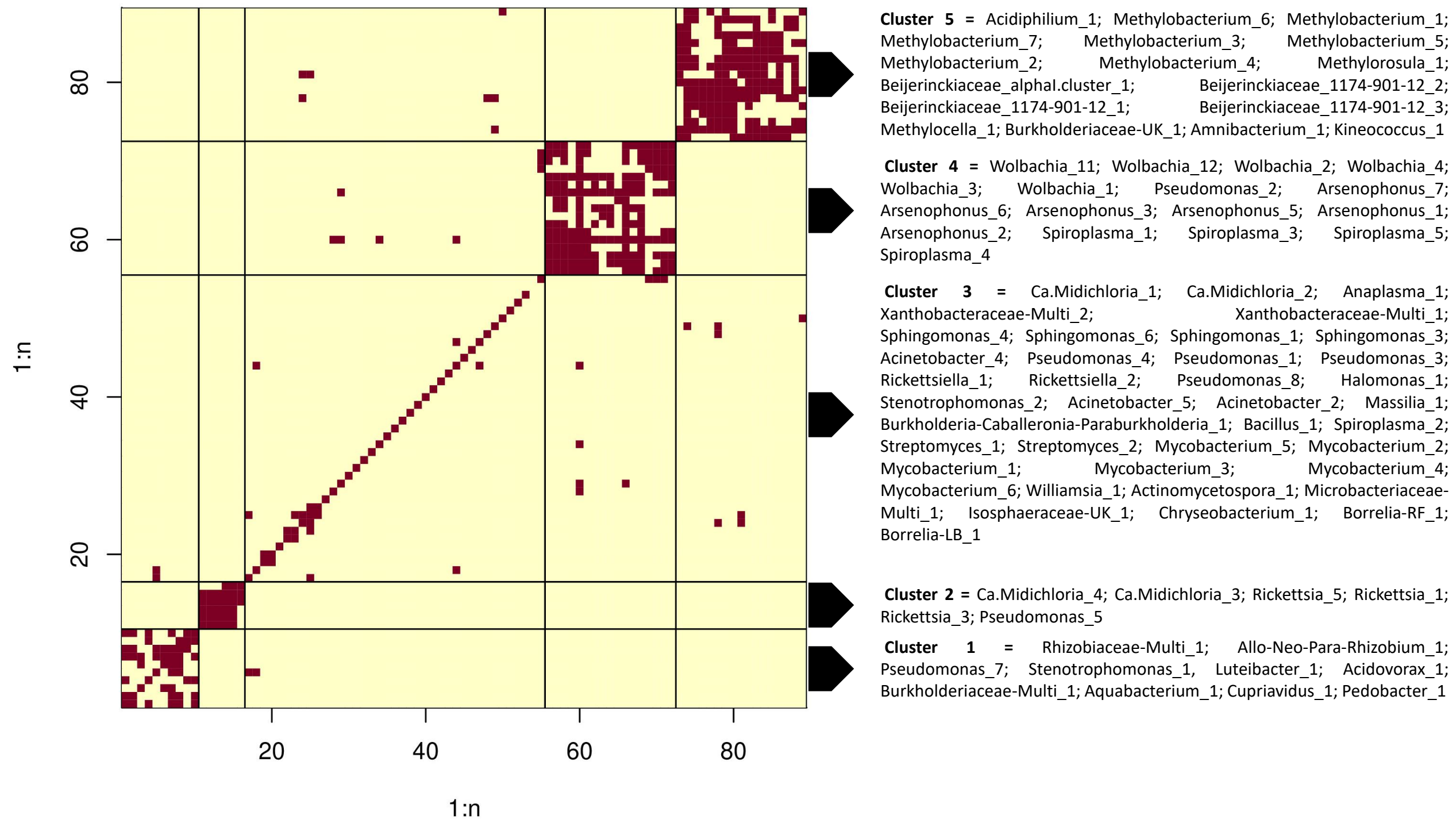

Supplement: Supplementary file 4 — Additional file 3. OTUs clusters identified from the total network. OTUs presenting similar connection profiles were identified and assigned to different clusters according to a stochastic block model obtained from the corresponding binary adjacency matrix, obtained from the network inferred by PLN network, which places an edge for each non-null partial correlation (whether positive or negative). [file 40168_2021_1051_MOESM4_ESM.pdf]
